# Efficient generation of GGTA1-null *Diannan* miniature pigs using TALENs combined with somatic cell nuclear transfer

**DOI:** 10.1186/s12958-016-0212-7

**Published:** 2016-11-08

**Authors:** Wenmin Cheng, Heng Zhao, Honghao Yu, Jige Xin, Jia Wang, Luyao Zeng, Zaimei Yuan, Yubo Qing, Honghui Li, Baoyu Jia, Cejun Yang, Youfeng Shen, Lu Zhao, Weirong Pan, Hong-Ye Zhao, Wei Wang, Hong-Jiang Wei

**Affiliations:** 1 College of Animal Science and Technology, Yunnan Agricultural University, Kunming, 650201 China; 2 State Key Laboratory for Conservation and Utilization of Bio-Resources in Yunnan, Yunnan Agricultural University, Kunming, 650201 China; 3 Research Center of Life Science, Yulin University, Yulin, 719000 China; 4 Hunan Xeno Life Science Co., Ltd, Changsha, 410600 China; 5 Institute for Cell Transplantation and Gene Therapy, The Third Xiangya Hospital Central-South University, Changsha, 410013 China; 6 Key Laboratory of Animal Nutrition and Feed of Yunnan Province, Yunnan Agricultural University, Kunming, 650201 China

**Keywords:** GGTA1, TALENs, Cloning, Xenotransplantation, *Diannan* miniature pigs

## Abstract

**Background:**

α1,3-Galactosyltransferase (GGTA1) is essential for the biosynthesis of glycoproteins and therefore a simple and effective target for disrupting the expression of galactose α-1,3-galactose epitopes, which mediate hyperacute rejection (HAR) in xenotransplantation. Miniature pigs are considered to have the greatest potential as xenotransplantation donors. A GGTA1-knockout (GTKO) miniature pig might mitigate or prevent HAR in xenotransplantation.

**Methods:**

Transcription activator-like effector nucleases (TALENs) were designed to target exon 6 of porcine GGTA1 gene. The targeting activity was evaluated using a luciferase SSA recombination assay. Biallelic GTKO cell lines were established from single-cell colonies of fetal fibroblasts derived from *Diannan* miniature pigs following transfection by electroporation with TALEN plasmids. One cell line was selected as donor cell line for somatic cell nuclear transfer (SCNT) for the generation of GTKO pigs. GTKO aborted fetuses, stillborn fetuses and live piglets were obtained. Genotyping of the collected cloned individuals was performed. The Gal expression in the fibroblasts and one piglet was analyzed by fluorescence activated cell sorting (FACS), confocal microscopy, immunohistochemical (IHC) staining and western blotting.

**Results:**

The luciferase SSA recombination assay revealed that the targeting activities of the designed TALENs were 17.1-fold higher than those of the control. Three cell lines (3/126) showed GGTA1 biallelic knockout after modification by the TALENs. The GGTA1 biallelic modified C99# cell line enabled high-quality SCNT, as evidenced by the 22.3 % (458/2068) blastocyst developmental rate of the reconstructed embryos. The reconstructed GTKO embryos were subsequently transferred into 18 recipient gilts, of which 12 became pregnant, and six miscarried. Eight aborted fetuses were collected from the gilts that miscarried. One live fetus was obtained from one surrogate by caesarean after 33 d of gestation for genotyping. In total, 12 live and two stillborn piglets were collected from six surrogates by either caesarean or natural birth. Sequencing analyses of the target site confirmed the homozygous GGTA1-null mutation in all fetuses and piglets, consistent with the genotype of the donor cells. Furthermore, FACS, confocal microscopy, IHC and western blotting analyses demonstrated that Gal epitopes were completely absent from the fibroblasts, kidneys and pancreas of one GTKO piglet.

**Conclusions:**

TALENs combined with SCNT were successfully used to generate GTKO *Diannan* miniature piglets.

**Electronic supplementary material:**

The online version of this article (doi:10.1186/s12958-016-0212-7) contains supplementary material, which is available to authorized users.

## Background

The increasing life expectancy of humans has led to an increase in the number of patients suffering from chronic diseases and end-stage organ failure [1]. The number of organ donated cannot meet the demands of organ transplantation. Xenotransplantation (e.g., from pigs to humans) may resolve this problem [2]. Miniature pigs and humans have similar organ physiology and anatomy. Compared with non-human primates, miniature pigs present a decreased risk of cross-species disease transmission due to their greater phylogenetic distance from humans [3]. The *Diannan* miniature pig, a famous local variety, has unique advantages, including early sexual maturity, high birth rate and low full-grown body weight (compared with the Large White pig) [4]. Moreover, because of its high litter size, the cloning efficiency of *Diannan* miniature pigs was higher than those of 19 different donor cell types from other pigs [4]. Thus, these pigs can be considered an ideal source for human xenotransplantation.

However, before miniature pigs can be successfully used for xenotransplantation, the major obstacles of hyperacute rejection (HAR) and acute humoral xenograft rejection (AHXR) must be overcome [5]. The galactosyl-α (1,3) galactose (Gal) epitope is strongly expressed in porcine endothelium and mediates HAR. α1,3-Galactosyltransferase (GGTA1) is essential for the biosynthesis of glycoproteins. A null mutation of GGTA1 may thus prevent the expression of the Gal epitope on porcine tissues [6], and GGTA1 knockout (GTKO) pigs may mitigate or prevent HAR during xenotransplantation.

GTKO pigs were generated using traditional homologous recombination (HR), zinc-finger nuclease (ZFN) gene editing technologies and somatic cell nuclear transfer (SCNT) methods [6–10]. However, methods for producing gene-modified pigs are inefficient, time-consuming and labor-intensive [11, 12]. TALEN is a versatile genome editing tool that has been successfully used for genome editing in various species. Several genetically modified embryos/pigs have been generated by TALENs, including mono- and biallelic mutations of the low-density-lipoprotein receptor gene [13], azoospermia-like and adenomatous polyposis coli gene knockout [14], polymorphic sequence variation within the transactivation domains of RELA [15] and CMAH knockout preimplantation embryos production [16]. These studies demonstrate the successful application of TALENs in pigs for efficient gene targeting. Another recently developed efficient genome editing tool, the clustered regularly interspersed short palindromic repeats (CRISPR)/CRISPR-associated 9 system (CRISPR/Cas9), is easier to employ and permits multiplexible targeting. Although CRISPR/Cas9 has been successfully developed and effectively used for genomic editing in a range of species [17–21], TALENs are more precise and have fewer pronounced off-target effects [22]. Therefore, we used TALENs to modify GGTA1 in porcine fibroblast to produce GTKO pigs via SCNT.

In this study, we aimed to efficiently generate GTKO fetuses and piglets using TALEN and SCNT technologies. We established the first genetically modified *Diannan* miniature pigs and performed a systematic phenotypic characterization of GTKO fibroblasts and *Diannan* miniature piglets. These GTKO miniature pigs might be ideal organ donors with the prevention of HAR and AHXR for xenotransplantation.

## Methods

### Chemicals

All of the chemicals were purchased from Sigma Chemical Co. (St. Louis, MO, USA) unless otherwise stated.

### TALEN design and generation

**Fig. 1:**
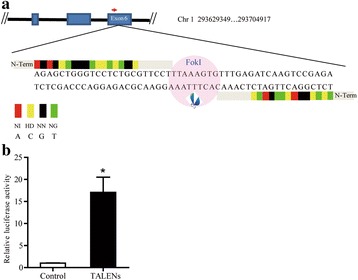
Schematic of TALENs targeting the porcine GGTA1 locus and the activity assay. **a** Schematic diagram of pig GGTA1 partial protein coding region and the TALENs targeting loci. The red arrow indicates the target site of the TALENs on the exon. **b** The SSA recombination assay was used to determine the targeting efficiency of the TALEN vector in vitro (*P <0.05)

TALENs targeting exon 6 of the porcine GGTA1 gene were designed and assembled by ViewSold Biotech (China, Beijing) (Fig. 1a). A luciferase single strand annealing (SSA) recombination assay was employed to evaluate the targeting efficiency of TALEN vectors in vitro using a specific method described previously [23]. In brief, 293 T cells in 24-well plates were transfected with 200 ng of TALEN expression plasmids, 50 ng of SSA reporter plasmid and 10 ng of Renilla plasmid. Each experiment was performed in triplicate. The cells were harvested 1 d after transfection and were treated with Luciferase Cell Lysis Buffer, followed by detection of relative luciferase activity.

### Cell culture, transfection and selection

Pig fetal fibroblasts (PFFs) were prepared as previously described [24]. In brief, PFFs were isolated from 35-day-old *Diannan* miniature pig fetuses and were digested. After centrifugation and re-suspension, the PFFs were cultured in a flask for 12 h and then frozen in DMEM supplemented with 20 % FBS and 10 % dimethyl sulfoxide for future use. The day before transfection, the PFFs were thawed and cultured in medium. Approximately 7 × 10^5^ PFFs in 700 μl PBS containing 21 μg of the TALEN plasmid pair were electroporated at 250 V for 20 ms with a Gene Pulser Xcell electroporator (Bio-Rad, California, USA). After electroporation, the cells were plated into T25 flask for 2 days in DMEM. The cell colonies were seeded individually into 48-well plates to isolate single colonies. Single cell-derived colonies were harvested after 12-14 d of culture, and the colonies were genotyped by PCR, T7 endonuclease I assay (T7EI) and sequencing.

### Oocyte collection and culture

Oocyte collection and culture were performed as previously described [24]. Ovaries were collected from Hongteng slaughterhouse (Chenggong Ruide Food Co., Ltd, Kunming, Yunnan Province, China). Cumulus-oocyte complexes (COCs) were aspirated from 3–6 mm diameter follicles. COCs with at least three layers of compacted cumulus cells were selected, and approximately 50 COCs were cultured in 200 μl IVM (in vitro maturation) medium [24] at 38.5 °C in an atmosphere with 5 % CO_2_ (APC-30D, ASTEC, Japan) and saturated humidity for 42–44 h.

### SCNT and generation of GTKO piglets

After IVM, COCs with expanded cumulus cells were briefly treated with 0.1 % (w/v) hyaluronidase, and the cumulus cells were removed by gently pipetting. The denuded oocytes were enucleated by aspirating the first polar body and adjacent cytoplasm using a beveled pipette in TLH-PVA. The cells identified as biallelic GTKO by gene sequencing were digested with trypsin and used as donor cells, which were injected into the perivitelline space of oocytes. Donor cells were fused with recipient cytoplasts in fusion medium using a single direct current pulse of 200 V/mm for 20 μs with an embryonic cell fusion system (LF 201, Nepa Gene Co. Ltd., Tokyo, Japan). The reconstructed embryos were cultured for 2 h in porcine zygote medium-3 (PZM-3) and then activated with a single pulse of 150 V/mm for 100 μs in an activation medium [24]. The reconstructed embryos were equilibrated in PZM-3 supplemented with 5 μg/ml cytochalasin B for 2 h at 38.5 °C in humidified atmosphere of 5 % CO_2_, 5 % O_2_ and 90 % N_2_ (APM-30D, ASTEC, Japan). Then, embryos were washed three times and cultured in PZM-3 medium under the same conditions described above. Cleavage and blastocyst rates were documented on day 2 and day 7, respectively.

Crossbred prepubertal gilts (Large White/Landrace Duroc) weighing 100 to 120 kg were used as surrogates for the cloned embryos. They were checked for estrus at 09:00 and 18:00 h daily. Reconstructed embryos cultured for 2 h after activation were surgically transferred to the oviducts of the surrogate. Pregnancy was detected approximately 23 days after embryo transfer using an ultrasound scanner (HS-101 V, Honda Electonics Co. Ltd., Yamazuka, Japan).

### Genotyping

A single-cell colony was selected for genotyping. Cell lysis was performed in 10 μl of NP-40 solution for 15 min at 65 °C and 10 min at 95 °C. Then they were used as templates for PCR amplification. The targeted fragments were amplified by PCR with specific primers (Additional file 1: Table S1) and then purified using a PCR cleanup kit (AP-PCR-50, Axygen, New York, USA). The purified PCR product mixture (50 ng of the wild-type PCR product added to 50 ng of the GGTA1-targeted PCR product) was denatured and reannealed in NEBuffer 2 (NEB, Massachusetts, USA) using a thermocycler. The PCR products were digested with T7ENI (M0302 L, NEB, Massachusetts, USA) for 30 min at 37 °C and separated by electrophoresis in a 1 % agarose gel. PCR products in which mutations were detected by the T7ENI cleavage assay were sub-cloned into a T vector (D103A, Takara, Dalian, China) for sequencing.

We also extracted genomic DNA from one live fetus, aborted fetuses and piglets for gene typing. The targeted fragments were amplified as described above and cloned into a T vector for sequencing. For each sample, colonies were selected randomly and were sequenced using M13F primer (Additional file 1: Table S1).

### Flow cytometric analysis

Fibroblasts (GTKO) derived from one two-month-old piglet were used for flow cytometric analysis. 293T cells were used as a negative control, and fibroblasts derived from *Diannan* miniature pigs cloned from unmodified GGTA1 PFFs by SCNT were used as a positive control. The cells were washed three times with PBS, stained with 20 μg/ml FITC-GS-IB4 lectin for 5 min at 37 °C, washed twice and re-suspended in 300 μl of PBS, and analyzed using a BD Accuri C6 flow cytometry (BD, New Jersey, USA).

### Fluorescent microscopy

Fibroblasts (GTKO), the negative control (293T) and the positive control were cultured on coverslips for 24 h, fixed with 4 % paraformaldehyde for 10 min, and washed with PBS. First, the cells were incubated in 0.2 % Triton X-100 for 10 min at room temperature and washed with PBS. The cells were then blocked with 1 % bovine serum albumin (BSA) in PBS (blocking buffer) for 1 h at room temperature and incubated overnight in a humid chamber at 4 °C with 40 μg/ml FITC-GS-IB4 in blocking buffer. The slides were washed with PBS, and the nuclei were counterstained with 1 μg/ml DAPI. The slides were covered with mounting medium and observed under a laser scanning confocal microscope (OLYMPUS FV 1000, Tokyo, Japan).

### Immunohistochemical analysis of tissue sections

Two-month-old GTKO pigs and SCNT cloned pigs from TALEN-unmodified donor fibroblasts were euthanatized by CO_2_ inhalation, and their kidneys were excised. Kidney sections were placed in a mold, and a small amount of OCT (optimal cutting temperature) was added to cover the tissue. The frozen blocks were stored at -80 °C until use. The tissues were then equilibrated to the temperature of the cryostat (-20 °C) and cut to the desired thickness (usually 5 μm). Tissue sections were fixed in 4 % paraformaldehyde and washed with PBS for three times. The slides were incubated in 3 % H_2_O_2_ and methanol solution for 30 min, then washed with PBS for three times, and dried. The slides were blocked with 5 % BSA in PBS for 15 min at room temperature in a humidified chamber. The tissue sections were then incubated with 5 μg/ml anti-gal antibody (ALX-801–090, Abcam, London, UK) at 4 °C overnight. After washing with PBS, the tissue sections were incubated with biotinylated antibody from an IHC kit (KIT-9901, Elivision TM plus Polyer HRP IHC Kit, Fuzhou, China) and stained using DAB (3,3′-diaminobenzidine).

#### Protein extraction and immunoblotting

Protein extraction and immunoblotting were performed as previously described in our previous study [25]. The pancreas tissue from GTKO piglets and cloned piglet derived from unmodified original donor cells were used to evaluate GGTA1 protein levels using western blotting. In brief, pancreas tissues were lysed in RIPA lysis buffer (Bestbio, China) with protease inhibitors at 4 °C. After lysis, the supernatants were obtained by centrifugation at 13,800 × g for 15 min at 4 °C. Equal amounts of protein (70 μg) were run on SDS-PAGE gel, along with molecular weight marker. After electrophoresis, the proteins were transferred to PVDF membranes and reacted with primary antibodies against GGTA1 (ALX-801-090-1, Enzo, Lausen, Switzerland; 1:15) and β-actin (anti-β-actin, Sigma-Aldrich; 1:2000) at 4 °C overnight. After incubation, the membranes were washed and incubated with anti-mouse secondary antibodies (R&D, USA). The membranes were incubated with the ECL (Easysee Western Blot Kit, China) and visualized with an Imaging System (Bio-Rad, Universal Hood II, USA).

### Statistical analysis

All of the data were expressed as the mean ± standard error (SE). *t*-test was performed using the SPSS 22.0 software package (IBM Crop, Armonk, NY). Statistical significance was defined as *P* < 0.05.

## Results

### TALENs activity validation

The activity of the designed TALEN targeting GGTA1 exon 6 was determined in vitro by using a luciferase single-strand annealing (SSA) recombination assay. The luciferase activity of the TALENs was 17.1-fold higher than that of the control (Fig. 1b).

### Generation of GTKO piglets using TALENs

**Fig. 2:**
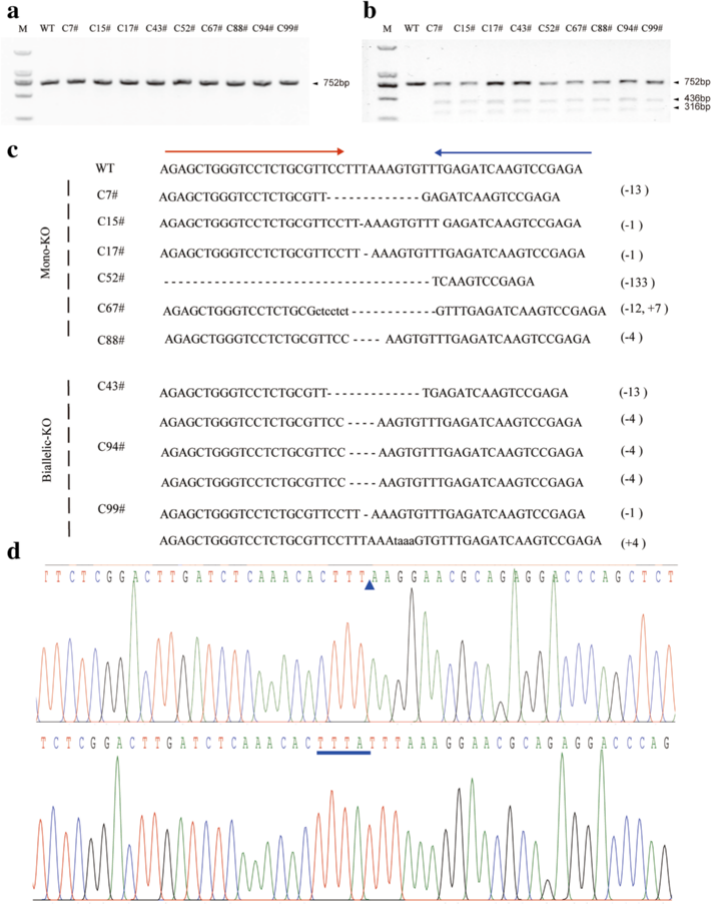
TALEN-mediated GGTA1 mutations in PFFs. **a** PCR product from the TALEN target locus in GGTA1-modified cell lines. **b** Detection of the *GGTA1* gene in cell colonies by PCR. The genomic regions surrounding the target site were amplified and a 752-base-pair PCR product of the *GGTA1* gene was obtained. **c** Genotyping of *GGTA1-*mutant cell lines by the T7EI assay. The *GGTA1* gene of each cell colony was assayed and presented in the same order as the PCR results. Individuals with one band of the wild-type (WT) and mutated alleles show three bands in the T7EI assay. **d** Representative sequencing chromatographs of the complementary sequence to the TALEN target site in C99# GTKO cell line

Nine cell colonies of the 126 single-cell colonies had modifications at the targeted site of GGTA1, and 3 of these colonies were biallelic GTKO (C43#, C94#, C99#) (Fig. 2). C99# GTKO cell colony was used as the donor cells for SCNT. We produced 2068 reconstructed embryos by SCNT, and the cleavage and blastocyst formation rates of the embryos were 75.2 % (1667/2068) and 22.3 % (458/2068), respectively (Table 1).

**Table 1:** Developmental competence of reconstructed embryos after fusion and electrical activation

No. reconstructed embryos	Cleaved (%)	Blastocysts (%)
2068	1667 (75.2 ± 4.2)	458 (22.3 ± 1.5)

**Fig. 3:**
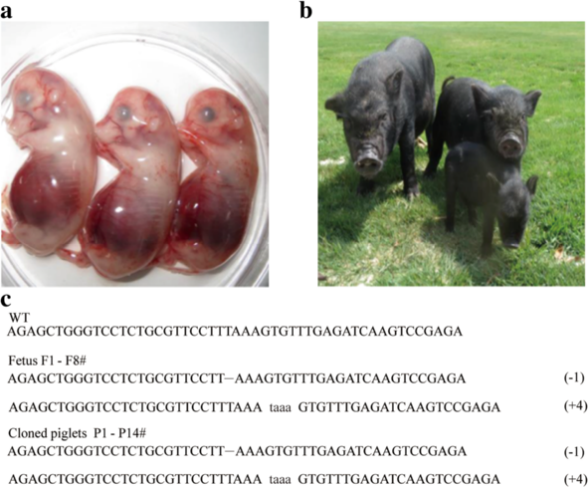
Cloned piglets. **a** Aborted GTKO fetuses after 42 days. **b** Newborn GTKO piglets. **c**. Sequences of the GGTA1 mutations in cloned fetuses and cloned piglets

The reconstructed GTKO embryos were transferred to 18 recipient gilts. There are 12 recipient gilts became pregnant and 6 miscarried with the yielding of 8 fetuses (Fig. 3a). One live fetus was obtained on the 33th day of gestation for genotyping. A total of 12 live (Fig. 3b) and two stillborn (Table 2) piglets were collected from 6 surrogates by either caesarean or natural birth.

**Table 2:** Development of reconstructed GTKO cloned embryos after transfer to recipient gilts

Recipients	Pregnancy	Days of pregnancy	No. of fetuses (Dead)	Offspring (stillborn)
1	+	33 (Cesarean)	1	
2	+	42 (Abortion)	3(3)	
3	+	117		2
4	-			
5	-			
6	-			
7	-			
8	-			
9	+	30 (Abortion)	3(3)	
10	+	114 (Cesarean)		3
11	+	120(Cesarean)		1
12	+	-		-
13	+	116 (Cesearean)		5(1)
14	+	28 (Abortion)	1(1)	
15	+	28 (Abortion)	1(1)	
16	+	115 (Cesarean)		2
17	-			
18	+	115		1(1)
Total	12 (66.7 %)	-	9(8)	14(2)

+ indicated pregnancy; - indicated not pregnancy

**Fig. 4:**
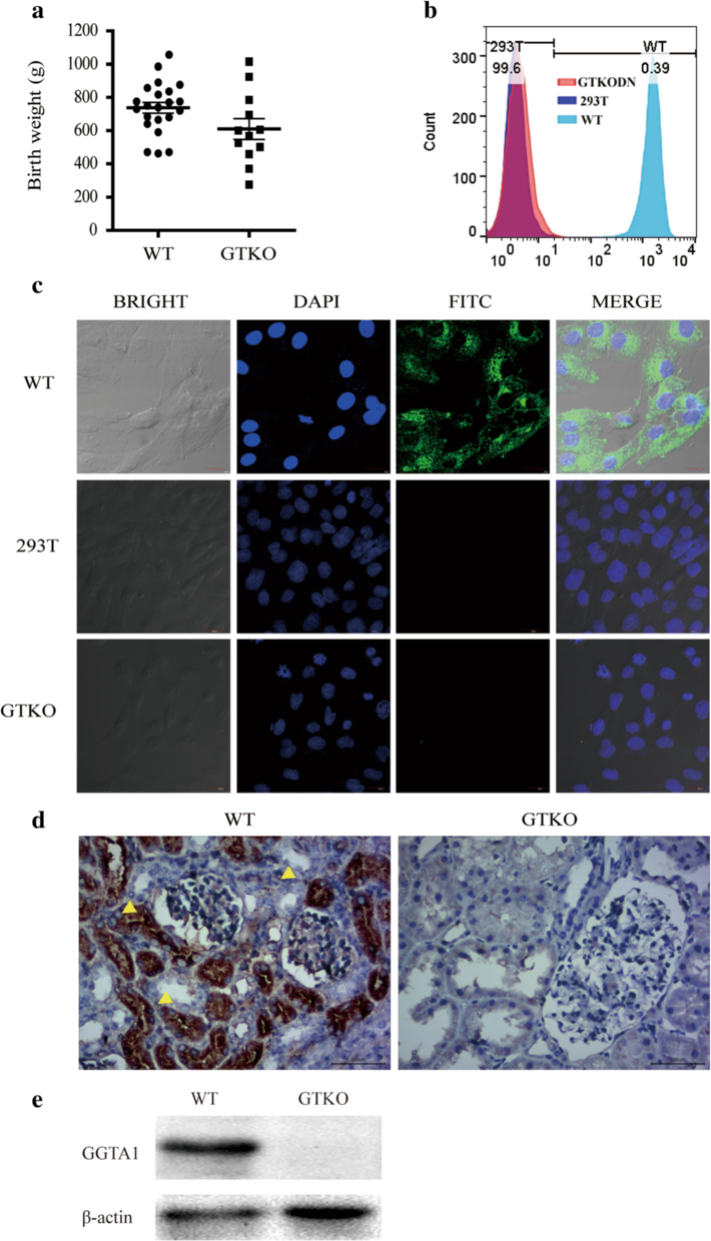
Phenotype detection. **a** Comparison of birth weight between cloned GTKO piglets and the control. **b** Flow cytometric analysis of GTKO pigs with FITC-conjugated GS-IB4 lectin staining. **c** Confocal microscopy analysis of fibroblasts from GTKO piglets stained with FITC-conjugated GS-IB4. **d** Immunochemical analysis of the GTKO pig kidney. Wild-type *Diannan* miniature pigs were used as the positive control. **e** Protein expression levels were assessed via Western blotting. GGTA1 protein expression in the pancreas tissue of GTKO and WT pig are shown in cropped blots using an anti-GGTA1 monoclonal antibody. Anti-β-actin served as a loading control

Sequencing analysis of the target site in all fetuses and piglets confirmed the homozygous GGTA1-null mutation, consistent with the genotype of the C99# donor cells (Fig. 3c). The average birth weight of the GTKO piglets (600 g) was slightly lower than that of the wild type control piglets (730 g) (Fig. 4a).

### Phenotype of the GTKO newborn piglets

Next, phenotype of the GTKO fibroblasts and newborn piglets were evaluated with various technologies. Compared with wild type positive control samples, the expression of Gal epitope was absent in both GTKO cells and negative control samples (Fig. 4b). Same results were obtained by using confocal microscopy: Gal epitope only expressed in the wild type positive control cells; while there is no Gal epitope expression in the GTKO cells and negative control cells (Fig. 4c). IHC analysis also confirmed the absence of Gal epitope expression in the kidneys of the GTKO piglets (Fig. 4d). Western blotting analysis demonstrated that GGTA1 protein expression in pancreas tissue of GTKO piglets was completely absent in the comparison of the expression in wild type control piglets (Fig. 4e). These results suggest that the GGTA1 gene had been successfully knocked out in the *Diannan* miniature pigs.

## Discussion

Animal-to-human organ transplantation (xenotransplantation) techniques would generate an unlimited supply of organs and tissues for the treatment of end-stage organ failure. Although non-human primates are closely related to humans, their smaller size, slow growth rates, limited production of offspring and difficulty of breeding in captivity limit their use as donor animals for xenotransplantation [26]. Pigs present several advantages over non-human primates and thus may serve as a large pool of animal donors for xenotransplantation in the future. One essential question regarding xenotransplantation is whether animal organs can serve as an effective physiological proxy for human organs [27]. The body weight of miniature pigs is typically less than 50 kg [28, 29], equivalent to that of an immature domestic pig. Therefore, compared with larger domestic pigs, miniature pigs are generally easier to handle and more suitable for medical application [30]. Among them, the cloned *Diannan* miniature pigs had been produced and suitable for further genetic modification [4].

TALENs have been used as genome editing tools to generate GGTA1-mutant pigs [31, 32]. By using limiting dilution method for the GTKO cell colonies’ selection, we successfully obtained three GGTA1 biallelic knockout colonies and produced the cloned piglets with the expected genotype from one GTKO cell colony. Compare with various methods for the selection of GGTA1-mutant somatic cells such as G418 selection [31] or IB4 lectin combined with magnetic beads selection [32], the efficiency of our method for GTKO cell colonies’ selection was slightly low. Furthermore, using TALEN mRNA [32] rather than TALEN DNA plasmids could increase the efficiency of GGTA1-mutant somatic cell selection. Therefore, either using alternative GTKO cell colonies’ selection methodology or using TALEN mRNAs might help to increase our efficiency for obtaining TALEN-mediated biallelic knockout cells. Moreover, our efficiency of generating GTKO piglets was slightly higher than that of previous studies [6–9, 31]. Our results suggested that this methodology was useful to produce the GTKO piglets. It has been reported that TALEN system exhibit high targeting specificity with little off-target effect [33, 34]. Previous similar in vivo studies of TALEN plasmid DNA editing in mammals like pig [35], mouse [36], monkey [37] did not observed detectable off-target effect either. Furthermore, our previous study also showed TALEN plasmid DNA editing in sheep [25] did not observed detectable off-target using whole-genome sequencing. These results suggest that TALEN plasmid DNA editing in *Diannan* miniature pig also have no off-target.

Although our system efficiently generated GGTA1-modified pigs, high abortion rates (33.37 %, 4/12) were observed. Abortion and fetal reabsorption were also observed in previous reports on GGTA1 knockout pigs and the reasons for these losses are unknown [9, 31]. GGTA1 encodes a member of the galactosyltransferase family of intracellular membrane-bound enzymes, which are involved in the biosynthesis of glycoproteins and glycolipids. The encoded protein catalyzes the transfer of galactose from UDP-galactose to N-acetyllactosamine in an α(1,3)-linkage to form galactose alpha(1,3)-galactose. There is no evidence that GGTA1 is involved in fetal development and growth, and no reports indicate that GGTA1 mutations induce the death of cloned animals. Therefore, the incomplete reprogramming of somatic cells in SCNT might be the reason for the observed abortion and fetal reabsorption. Stillborn piglets are another barrier to the efficient generation of live GGTA1-modified piglets. Our previous study showed that the stillborn piglets would have survived if caesarean sections had been performed prior to full gestation [4]. Therefore, caesarean sections were performed to aid the delivery of surrogate pigs to improve the survival rates of the cloned piglets in the present study. In this study, the higher piglet survival rate (7/8) achieved by caesarean section compared with that natural birth (2/3) also supports our previous result.

The primary purpose of generating GTKO pigs is to overcome the primate humoral response [6, 8], and these pigs are considered a platform for testing existing and future genetic solutions for xenotransplantation [38]. Even when immune, coagulative, and pro-inflammatory responses to grafts can be successfully overcome, the long-term graft survival and the functionality of transplanted pig organs and/or cells in a foreign environment is still unknown [39]. We have heterotopically transplanted the heart and one kidney from a GTKO pig into a Crab-eating Macaque. HAR did not occur in the Crab-eating Macaque, and the transplanted heart and kidney restored normal function. The heart began to beat and the kidney began to facilitate urination in the Crab-eating Macaque (date not shown). These results suggest that modified pigs have great potential in terms of reduced injury to pig organs following transplantation into non-human primates. In addition, previous investigations have demonstrated that the absence of galactose-α-1,3-galactose expression reduces the human T-cell proliferative response and cytokine responses [40]. However, this reduction cannot sufficiently reduce the requirement for exogenous immunosuppressive therapies to permit clinical use. Therefore, further genetic modifications of pigs are likely necessary [2].

## Conclusions

The combination of TALEN gene editing technology and SCNT is effectively used for the generation of biallelic GTKO *Diannan* miniature pigs. The rapid production of GTKO *Diannan* miniature pigs will enable many new applications in the future and help the development of xenotransplantation and alleviate the shortage of organs for clinical application.

## Additional file

Additional file 1: Table S1. GGTA1-targeted fragment PCR amplification primers and TA cloning sequencing primer. (DOC 29 kb)

## Abbreviations

AHXR: Acute humoral xenograft rejection
BMs: Bama miniature pigs
BSA: Bovine serum albumin
CMAH: Cytidine monophospho-N-acetylneuraminic acid hydroxylase
COCs: Cumulus-oocyte complexes
CRISPR/Cas9: Clustered regularly interspaced short palindromic repeats/ CRISPR associated 9
DAB: 3,3′-diaminobenzidine
DAPI: 4′,6-diamidino-2-phenylindole
DMEM: Dulbecco’s modified Eagle’s medium
FACS: Fluorescence activated cell sorting
FBS: Fetal bovine serum
FITC: Fluorescein isothiocyanate
Gal: Galactose
GGTA1: α1,3-galactosyltransferase
GTKO: GGTA1 knockout
HAR: Hyperacute rejection
HR: Homologous recombination
IVM: in vitro maturation
OCT: optimal cutting temperature
PCR: Polymerase chain reaction
PFFs: Pig fetal fibroblasts
PZM-3: Porcine zygote medium-3
RELA: v-rel reticuloendotheliosis viral oncogene homolog A
SCNT: Somatic cell nuclear transfer
SE: Standard error
SPSS: Statistical Product and Service Solutions
SSA: Single-strand annealing
TALENs: Transcription activator-like effector nucleases
TBs: Tibetan miniature pigs
TLH-PVA: HEPES-buffered Tyrode’s medium containing polyvinylalcohol
UDP-galactose: Uridine diphosphate- galactose
ZFN: Zinc-finger nuclease
