# Supplementary material for: Efficient generation of GGTA1-null Diannan miniature pigs using TALENs combined with somatic cell nuclear transfer
Source: Reprod Biol Endocrinol. 2016 Nov 8;14:77. doi: 10.1186/s12958-016-0212-7 (PMC5100250; doi:10.1186/s12958-016-0212-7)
Supplement: Additional file 1: Table S1. — GGTA1-targeted fragment PCR amplification primers and TA cloning sequencing primer. (DOC 29 kb) [file 12958_2016_212_MOESM1_ESM.doc]

**Table S1** *GGTA1* targeted fragments PCR amplifation primers and TA cloning sequencing primer

| Name | Sequence (5’ to 3’) |
| --- | --- |
| GGTA-F | 5’-TGAATGACTGAATGGTGGAGAG-3’ |
| GGTA-R | 5’-GTGGGTTTGTTGAGAAGGAAATAC-3’ |
| M13F | 5'-CGCCAGGGTTTTCCCAGTCACGAC-3' |
